# Protective Role of Silymarin on Hepatic and Renal Toxicity Induced by MTX Based Chemotherapy in Children with Acute Lymphoblastic Leukemia

**DOI:** 10.4084/MJHID.2016.043

**Published:** 2016-09-01

**Authors:** Adel A. Hagag, Mohamed A. Elgamsy, Hassan M. El-Asy, Maaly M. Mabrouk

**Affiliations:** 1 Pediatrics, Faculty of Medicine, Tanta University; 2 Clinical Pathology, Faculty of Medicine, Tanta University

## Abstract

**Background:**

ALL is the most common childhood malignancy. The children with ALL are treated with methotrexate (MTX) based chemotherapy protocols. MTX causes unpredictable serious hepatic and renal side effects. Silymarin has antioxidant and anti-inflammatory activities and stimulates tissue regeneration. This study aims to evaluate the protective effects of Silymarin on MTX-based chemotherapy-induced Hepatic and renal toxicity in children with ALL.

**Patients and Methods:**

80 children with newly diagnosed ALL were enrolled in the study. They were randomly divided into two groups. *Group I* included 40 children with ages ranging from 4–13 years and the mean age of 6.85± 2.89 years, who received Silymarin 420 mg/day in 3 divided doses for one week after each MTX dose. *Group II* included 40 children, with ages ranging from 4–12 years and the mean age of 7.30±2.6 years, who received placebo for one week after MTX therapy. For all patients liver functions including serum bilirubin, total proteins, albumin, globulin and albumin-globulin ratio, alkaline phosphatase, ALT and AST, prothrombin time and activity and renal functions including blood urea and serum creatinine, serum cystatin C and urinary N-acetyl-beta-D-glucosaminidase were done to assess hepatic and renal toxicity before and after chemotherapy.

**Results:**

There were no significant differences between group I and II as regard liver and renal functions before chemotherapy. After chemotherapy, there were significantly higher values of ALT and AST and alkaline phosphatase, and significantly lower Prothrombin activity in group II compared with group I. No significant differences between group I and II were found in total bilirubin, serum protein, and albumin levels. There was significantly lower blood urea, serum creatinine, and cystatin C and urinary N-acetyl-beta-D-glucosaminidase in group I compared with group II.

**Conclusion:**

Silymarin improved some hepatic and renal functions in children with ALL who received MTX-based chemotherapy protocols. Recommendations: Extensive multicenter studies could be recommended to prove the hepatic and renal protective effects of Silymarin in patients with ALL who received MTX-based chemotherapy protocols.

## Introduction

Acute Lymphoblastic Leukemia (ALL) is the most common childhood malignancy, representing one-third of pediatric cancers.^1^

Methotrexate (MTX) is a key drug in the curative regimen of children with ALL.^2^ MTX is a potent hepatotoxic agent that causes injury of the liver associated with impaired liver functions.^3^ The underlying mechanism of MTX hepatotoxicity remains unclear, however, MTX causes oxidative stress in liver tissue as it is metabolized and stored in hepatocytes in a polyglutamates form which delays the clearance of MTX providing a depot that extends the duration of exposure to the drug.^4^

Methotrexate is also nephrotoxic due to precipitation of MTX or its metabolites in the renal tubules causing obstruction and diminution of renal clearance with a consequent prolongation of MTX high levels. High MTX levels may, in turn, lead to an ineffective rescue by leucovorin and an enhancement of MTX toxicity. Also, MTX and its metabolites are relatively insoluble in acid urine.^5^

MTX hepatic and renal toxicity can lead to discontinuation or reduction of chemotherapy doses which may affect the overall prognosis, so it is essential to search for certain drugs to reduce MTX side effects especially hepatic and renal toxicities.^6^,^7^

Silymarin is an active extract from the seeds of the plant milk thistle (*Silybum marianum*) and is commonly known as Milk Thistle. The most prevalent component of the Silymarin complex is silybin or Silibinin (50–60% of silymarin) which is the most active photochemical and is widely responsible for benefits of silymarin. Besides silybin, considerable amounts of other flavonolignans are present in silymarin complex, namely silly Christin (20%), silydianin (10%), isosilybin (5%), dehydrosilybin, and a few flavonoids, mainly taxifolin.^8^,^9^ Silymarin has cytoprotective effects against several classes of hepatotoxic drugs like acetaminophen and galactosamine and nephrotoxic drugs like cisplatin;^10^ furthermore, a study of Ghaffari et al. 2011^11^ showed significantly lower ALT and AST levels in rats receiving MTX plus Silymarin than rats receiving MTX and placebo. The protective effects of orally administered Silymarin have also been demonstrated in patients with ALL under MTX-based chemotherapy.^12^,^13^

The purpose of the work was to study the protective effects of Silymarin on MTX-based chemotherapy-induced hepatic and renal toxicity in children with acute lymphoblastic leukemia.

## Subjects and Methods

This randomized study was conducted on 80 children with newly diagnosed ALL, admitted to Hematology Unit, Pediatric Department, Tanta University Hospital. It obtained the preventive approval from ethical committee of Research Centre in Tanta University and written consent from parents of enrolled children.

Randomization was done using sequentially numbered, opaque, sealed envelopes. So the patients were randomly divided into two equal groups:

### Group I

40 children with ALL including 24 males and 16 females with ages ranging from 4–12 years and the mean age of 6.85 ± 2.89 years. They were treated with methotrexate-based treatment protocol^14^–^18^ and delayed Ca leucovorin rescue according to methotrexate levels.^19^ Silymarin 420 mg/day, (In the form of Legalon- *MEDA Pharma GmbH-* tablets 140 mg per tablet one hour before each meal (3 times daily) or Hepaticum syrup (Medical Union Pharmaceuticals, Egypt) in case of inability to swallow the tablet form; (each 5 ml of Hepaticum contains 50 mg Silymarin) was added in 3 divided doses one hour before each meal for one week after each MTX dose.^7^,^20^

### Group II

40 children with ALL including 26 males and 14 females with their age ranging from 4–12 years and the mean age of 7.30 ± 2.69 years. They were treated with MTX based treatment protocol^14^–^18^ and delayed Ca leucovorin rescue according to MTX levels^19^ and placebo for one week after each methotrexate dose.

### Inclusion criteria

Children with newly diagnosed acute lymphoblastic leukemia who were treated with the methotrexate-based treatment protocol.

### Exclusion criteria

Patients with acute lymphoblastic leukemia with hepatitis A, B or C.

*All patients were subjected to a full history* taking, and clinical examination with a special account of fever, pallor, purpura, bone ache, hepatosplenomegaly, generalized lymphadenopathy and neurological features. Tools and laboratory investigations for diagnosis of ALL were performed according to clinical presentation. They always included complete blood count, BM aspiration with morphological, cytochemical smears and immunophenotyping and was based on the presence of ≥ 20% blast cells in BM according to WHO proposal and MPO negative staining and immunophenotyping consistent with ALL, spinal puncture with liquor examination.^21^–^24^

Bone marrow aspiration was done under complete aseptic technique. Smears of direct BM aspirate were prepared, stained with Lieshman stain for morphologic study and cytochemical stains with Sudan black and Myeloperoxidase. Immunophenotyping was performed using the following panel of fluorescein isothiocyanate/phycoerythrin-conjugated monoclonal antibodies: Lymphoid T-cell markers (CD2, CD3, CD5, CD7), Lymphoid B-cell markers (CD10, CD19, CD20, CD22) and Myeloid cell markers (CD13, CD33).^21^–^24^

### Liver and renal functions to assess methotrexate hepatic and renal toxicity before and after chemotherapy

A venous blood sample of 5.8 ml was collected from each patient and delivered into three tubes: 1 ml of 20 uL EDTA solution for complete blood count including differential WBCs which was done on Leishman stained peripheral blood smear with evaluation using ERMA PCE-210 N cell –counter from Erma, Inc. Japan;^25^ 1.8 ml blood into tube containing 0.4 ml sodium citrate for prothrombin time and activity;^26^ 3 ml into a plain tube for other liver functions including serum bilirubin, total protein, albumin, globulin and A/G ratio,^27^ serum alkaline phosphatase, alanine aminotransferase (ALT) and aspartate aminotransferase (AST)^28^ and renal functions including blood, urea serum creatinine and blood urea nitrogen (BUN),^29^ and serum cystatin C. Cystatin was measured by latex particle-enhanced turbidimetric immunoassay (PETIA) on Hitachi 7600 auto-analyzer (Hitachi Co., Tokyo, Japan) using HiSense kit (HBI Co., Anyang, Korea)^30^ and urinary N-acetyl-beta-D-glucosaminidase (NAG) which was estimated kinetically using Sigma reagents (USA).^31^ Serum for serum cystatin C and early morning urine samples for urinary NAG were collected, centrifuged, aliquoted and frozen at − 20 °C. Samples were thawed and mixed thoroughly just before the assay to avoid erroneous results of repeated freeze/thaw cycles.^32^

### Protocol of treatment used in the studied ALL patients

#### Induction (6 weeks)

IV Vincristine 1.5mg/kg/m^2^/week (days 0, 7, 14, 21, 28, 35), Doxorubicin 25mg/m^2^/week IV infusion (days 0, 7, 14, 21, 28, 35), asparginase 6000 u/m^2^ SC on alternate days (10 doses) and oral prednisone 40 mg/m^2^/day for 6weeks. On day 21, BM aspiration was done; If BM blast cells is more than 5 %, we add etoposide 100 mg/m2/dose IV (days 22, 25, 29), cyclophosphamide 750mg/m^2^/dose IV infusion (days 22, 25, 29), aracytin 100/m^2^/dose IV (days 22, 25, 29), and methotrexate 5g/m^2^ over 4 hours on day 28.^13^

#### Consolidation (9 weeks)

IV methotrexate 1gm/m^2^/dose over 24 hour infusion on days 0, 21, 42 and 63, oral mercaptopurine 60 mg/m^2^ daily on days 0–13 and 28–41, IV vincristine 1.5 mg/m^2^ on days 14, 21, 42 and 49, PEG asparaginase 2,500 units/m^2^ IM on days 14 and 22, cyclophosphamide 750 mg/m^2^/dose IV infusion on days 0 and 28, aracytin 100/m^2^/dose IV on days 1–4, 8–11, 29–32 and 36–39 and age-adjusted intrathecal methotrexate on days 1,8,15 and 22.^14^,^15^

#### Interim maintenance (6 weeks)

IV Vincristine 1.5 mg/m^2^ on days 0, 10, 20, 30, 40, IV methotrexate starting dose of 100 mg/m^2^/dose over 10–15 minutes on day 0 thereafter escalate by 50 mg/m^2^/dose on days 10, 20, 30 and 40, PEG asparaginase 2,500 units/m^2^ IM on days 1 and 21 and age-adjusted intrathecal methotrexate on days 0 and 30.^15^

#### Delayed–intensification (6 weeks)

Oral dexamethasone 10 mg/m^2^/day days 1–7 and 14–21, IV vincristine 1.5 mg/m^2^ on days 0, 7 and 14, IM or IV pegylated L-asparaginase 2500 u/m^2^ on day 4, doxorubicin 25 mg/m^2^ IV push on days 0, 7 and 14, IV cyclophosphamide 1gm/m^2^ over 30 minutes on day 28, oral 6-thioguanine 60 mg/m^2^ on days 28–41, aracytin 75mg/m^2^ on days 29–32 and 36–39 and age-adjusted intrathecal methotrexate on day 28.^14^

#### Maintenance (30 months)

Weekly IV methotrexate 20 mg/m^2^, prednisone 120 mg/m2/day for five days every three weeks, vincristine 2 mg/m^2^ IV every three weeks, oral 6-mercaptopurine 50 mg/m^2^/day for 14 days every three weeks and age-adjusted intrathecal methotrexate every 18 weeks.^17^

### Dose and duration of Ca-leucovorin

18 The groups I and II received the same doses and length of leucovorin rescue which was started 48 hours after the start of MTX infusion in dose of 10 mg/m^2^ orally or IV every 6 hours but adjusted after that according to the MTX levels in each patient separately as follows:

- If methotrexate level is < 0.5 μmol/L: Ca leucovorin 15 mg/m^2^ is given every 3 hours (4 doses) then15 mg/m^2^ every 6 hours (8 doses).
- If methotrexate level is 0.5–1 μmol/L: Ca leucovorin 30 mg/m^2^ is given every 3 hours until MTX level becomes less than 0.1 μmol/L then 15 mg/m^2^ every 6 hours (8 doses).
- If methotrexate level is > 1 μmol/L: Ca leukovorin 50–100 mg/m^2^ is given every 3 hours until MTX level becomes less than 0.1 μmol/L then 15 mg/m^2^ every 6 hours (8 doses).

### Statistics

The collected data were organized, tabulated and statistically analyzed using SPSS software statistical computer package version 13. For qualitative data, the comparison between two groups was made using Chi-square test (X^2^). For comparison between means of two different groups, parametric analysis (t-test) and non-parametric analysis (Mann-Whitney U test) were used. Significance was adapted at P< 0.05 for interpretation of results of tests of significance.^33^

## Results

There were no statistically significant differences between studied groups as regard age and sex. Pallor, purpura, hepatomegaly, and splenomegaly represent the main presenting clinical manifestations in the studied patients at the time of diagnosis with no significant differences between Group I and II regarding clinical presentations. (Table 1).

There were normocytic normochromic anemia, thrombocytopenia, leukocytosis and increased blast cells in peripheral blood in studied patients with no significant differences between Group I and II as regard parameters of complete blood count (Table 1).

There were no statistically significant differences between studied groups as regard immunophenotyping at the time of diagnosis (Table 1).

There were no statistically significant differences between Group I and II as regard liver functions before chemotherapy (Table 2).

There were significantly higher ALT, AST and alkaline phosphatase, significantly lower prothrombin activity and prolonged prothrombin time in Group II compared with Group I after MTX therapy; however serum bilirubin, total proteins, and albumin show no significant differences between Group I and II (Table 3).

There were no significant differences between Group I and II as regard kidney functions before chemotherapy while after MTX therapy, there was statistically significant reduction in blood urea and serum creatinine, serum cystatin C and urinary N-acetyl-beta-D-glucosaminidase in Group I compared with Group II (Table 4).

There were no significant differences between studied groups as regard of continuation of chemotherapy (Table 5).

## Discussion

Leukemias are the most common childhood malignancy.^34^ Methotrexate is widely used in the treatment of various malignancies and non-oncological diseases, and its use is considered of primary importance in the treatment of ALL, even if it is limited by its toxicity.^35^ Silymarin is a plant extract that has cytoprotective effects against several classes of hepatotoxic and nephrotoxic drugs.^35^

The aim of the present study was to evaluate the supposed protective effects of Silymarin on methotrexate-induced hepatic and renal toxicity in 80 children with ALL who randomly received, as a part of the treatment protocol, Silymarin (Group I) or a placebo (Group II).

In the current study, after MTX therapy the serum levels of ALT and AST were significantly lower in the group I compared with the group II. These data were in agreement with Ladas et al., 2010^11^ who found that the administration of a 28 days course of Silymarin was associated with a significant reduction in AST and a trend towards a reduction in ALT at Day 56 from the baseline, but not immediately after cessation of supplementation. The latency of reduction of AST and ALT could be due to delayed effects of milk thistle, inadequate dosing, or short duration of supplementation. In our study; there were significantly lower alkaline phosphatase levels in group I compared with group II. This datum parallels with experiments of Ghaffari et al. 2011^11^ who found significantly lower levels of alkaline phosphatase among rats receiving methotrexate and Silymarin compared to the group receiving Methotrexate only.

In the present study, there were significant differences between the studied groups as regard prothrombin time and activity with prolonged prothrombin time in group II compared with group I, but no available studies in the literature for comparison.

In the present study, there were no significant differences in total serum protein and albumin levels and A/G ratio between Group I and II after MTX therapy. This datum is in agreement with Al–Fakhri and Abid, 2005^13^ who, studying the effects of maintenance therapy with MTX and 6–mercaptopurine on the liver of 30 children with ALL, did not find significant changes in total serum proteins, albumin, and A/G ratio.^13^ In fact, the liver can increase protein and albumin biosynthesis during diseases associated with protein loss, or in the presence of liver cell damage or injury induced by cytotoxic drugs until severe parenchymal damage.^7^

In the present work, there were no significant differences as regard serum bilirubin between group I and II before and after chemotherapy. These results support the results of Ladas et al., 2010^11^ who studied the effect of Silymarin on liver toxicity in children with ALL during maintenance therapy. Their patients were randomized to receive Silymarin or placebo daily for 28 days starting on the day following chemotherapy. Hepatic toxicity was investigated at Day 0, Day 28, and Day 56 and they found no significant differences in mean serum bilirubin between silymarin and placebo groups. On the other hand Ghaffari et al., 2011^11^ studied the effects of Silymarin on hepatic fibrosis due to MTX in rat and found significantly lower serum bilirubin levels in Silymarin receiving group.

The improvement of hepatic functions in Silymarin group is in agreement with Neuman et al. 1999^37^ who studied the effect of cytochrome

P450 2E1-inducers on MTX-induced cytotoxicity in human hepatocytes, and the silymarin role in preventing this toxicity. The cells were exposed to MTX in the presence of either ethanol or acetaminophen, with or without Silymarin. Ethanol and acetaminophen increased MTX cytotoxicity 2.9 times and 1.9 times, by an increase in IL 6, IL 8 and TNF-alpha and reduce both cGSH and mGSH and thus causing oxidative stress. The addition of silymarin downregulated expression of TNF-alpha and reduced the release of cytokines and abolished this toxicity^37^, so showing an anti-inflammatory/anti-fibrotic effect.^38^

The hepatoprotective and antioxidant activity of silymarin concern its ability to inhibit the free radicals that are produced from the metabolism of toxic substances such as ethanol, acetaminophen, and carbon tetrachloride. The generation of free radicals is known to damage cellular membranes and cause lipoperoxidation. Silymarin enhances hepatic glutathione and may contribute to the antioxidant defense of the liver. It has also been shown that silymarin increases protein synthesis in hepatocytes by stimulating RNA polymerase I activity.^39^,^40^

The free radical scavenging properties of Silymarin is higher than other antioxidants as Vitamin E and C;^41^ this could prevent or reduce the onset and progression of chemotherapy-induced hepatotoxicity in patients with ALL.^8^ Silymarin can also stabilize all cell membranes as it interacts with cell membrane components to prevent any abnormalities in the content of lipid fraction responsible for maintaining normal fluidity and can stimulate tissue regeneration and inhibit the deposition of collagen fibers.^41^

Silibinin, the most prevalent and active component of Silymarin complex, is able to stimulate DNA-dependent RNA polymerase I and increase rRNA synthesis, and this accelerates the formation of intact rRNA polymerase with resultant formation of new hepatocytes.^8^,^9^

In the present study, there were no significant differences between studied groups as regard kidney functions before chemotherapy while there was a significant reduction in renal MTX induced toxicity, evaluated throughout blood urea, serum creatinine, BUN, serum Cystatin C and urinary NAG in Group I compared with Group II.

Serum Cystatin C and urinary NAG provide an early and sensitive marker of occult tubular dysfunction resulting from renal disease or nephrotoxic damage. False positive results are rare, and its activity remains high during active disease or sustained toxic insult but falls to normal on recovery or removal of toxin.^32^,^42^

No human studies to compare with but this is in agreement with Dabak and Kocaman 201543 who studied the protective effect of Silymarin against MTX nephrotoxicity in Rats and found a significant reduction in MTX-induced renal damage in Silymarin group.

## Conclusion

Silymarin in the dose of 420 mg daily for one week after each dose of MTX improved some hepatic and renal functions in children with ALL who received MTX based chemotherapy protocols.

## Recommendations

Extensive multicenter studies on a large number of patients with longer follow-up duration and more advanced methods of assessment of hepatic and renal toxicity are required to confirm the protective effects of Silymarin against MTX-induced hepatic and renal toxicity in patients with ALL who received MTX based chemotherapy.

## Figures and Tables

**Table 1:** Comparison between studied groups as regard main clinical presentations and laboratory data at the time of diagnosis.

Clinical presentations		Group I (No=40)	Group II (No=40)	X^2^	P-value

**Age (years)**					
Range		4–13	4–12	0.259	0.614
Mean + SD		6.85 ± 2.89	7.30 ± 2.69		

**Sex**					
Male		24 (60%	26 (65%)	0.107	0.500
Female		16 (40%))	14 (35%)		

**Fever**		22 (55%)	20 (50%)	0.100	0.500

**Pallor**		31 (77.5%)	32 (80%)	0.025	0.653

**Purpura**		28 (70%)	30 (75%)	2.667	0.095

**CNS infiltration**		2 (5%)	2 (5%)	0.023	0.756

**Bone ache**		18 (45%)	16 (40%)	0.114	0.500

**Hepatomegaly**		38 (95%)	34(85%)	3.137	0.091

**Splenomegaly**		40 (100%)	36 (90%)	5.714	0.054

**Lymphadenopathy**		28 (70%)	26(65%)	1.758	0.160

**Immunophenotyping**					
Early pre-B-ALL		30	28	X^2^ 3.034	0.219
Pre-B-ALL		6	10		
T-ALL		4	2		

Hb (g/dl)	Range	5.9 – 11.5	3 – 12.9	3.105	0.086

	Mean ± SD	7.92 ±1.86	6.69 ± 2.49		

MCV(fL)	Range	70 – 99	70 – 99	1.249	0.271

	Mean ± SD	81.81 ± 7.57	84.55 ± 7.96		

MCH (pg)	Range	26 – 31	28 – 33	1.136	0.293

	Mean ± SD	29.41 ± 1.34	29.9 ± 1.59		

MCHC (%)	Range	29 – 34.5	31 – 35	1.647	0.207

	Mean ± SD	31.9 ± 1.66	32.5 ± 1.28		

WBCs (10^3^/mm^3^)	Range	1.9 – 121	2.7 – 130	1.997	0.166

	Mean ± SD	28.61 ± 86.63	32.15 ± 90.07		

Platelets (10^3^/mm^3^)	Range	20 – 293	10 – 191	1.426	0.240

	Mean ± SD	83.7± 63.92	72.3 ± 48.33		

Peripheral blood Blast cells %	Range	0 – 86	0 – 90	4.124	0.09

	Mean ± SD	37.25 ± 24.85	40.8 ±27.82		

CNS: Central Nervous System. Hb: Hemoglobin, WBCS: White blood cells, MCV: mean corpuscular volume, MCH: mean corpuscular hemoglobin, MCHC: mean corpuscular hemoglobin concentration.

**Table 2:** Comparison between studied groups as regard liver functions before the start of chemotherapy.

Parameters		Group I (No=40)	Group II (No=40)	t. test	P. value
Serum ALT (U/L)	Range	13–77	16–87	2.766	0.104
	Mean ± SD	24.35 ± 15.76	35.10 ± 14.52		
Serum AST (U/L)	Range	17–57	14–64	3.609	0.065
	Mean ± SD	23.95 ± 11.76	27.25 ± 19.58		
Total serum Bilirubin (mg/dl)	Range	0.42 – 1.2	0.4 – 0.9	0.323	0.573
	Mean ± SD	0.76 ± 0.2	0.73 ± 0.14		
Direct bilirubin (mg/dl)	Range	0.15 – 0.4	0.11 – 0.3	1.417	0.241
	Mean ± SD	0.26 ± 0.08	0.24 ± 0.05		
Indirect bilirubin (mg/dl)	Range	0.20 – 0.8	0.28 – 0.7	0.100	0.754
	Mean ± SD	0.49 ± 0.17	0.48 ± 0.15		
Alkaline phosphatase (U/L)	Range	112–209	109 – 219	0.118	0.734
	Mean ± SD	155.40 ± 31.24	151.65 ±37.63		
Albumin (g/dl)	Range	3.1 – 4.3	3–4.7	0.033	0.858
	Mean ± SD	3.79 ± 0.34	3.77 ± 0.52		
Total protein (g/dl)	Range	6–8.3	5.4 – 8.3	4.127	0.059
	Mean ± SD	7.17 ± 0.68	6.93 ± 0.69		
Albumin/Globulin ratio	Range	0.9 – 2.1	0.9 – 1.5	0.169	0.683
	Mean ± SD	1.19 ± 0.26	1.22 ± 0.15		
Prothrombin time (seconds)	Range	12–18.6	12.6 – 21	2.269	0.140
	Mean ± SD	14.54 ± 1.8	15.54 ± 2.34		
Prothrombin activity (%)	Range	70 – 100	60 – 100	0.958	0.334
	Mean ± SD	88.75 ± 9.3	85.5 ±11.57		

ALT: Alanine transferase, AST: Aspartate transferase.

**Table 3:** Comparison between studied groups as regard liver functions after chemotherapy.

Parameters		Group I (No=40)	Group II (No=40)	t. Test	p. value
Serum ALT (U/L)	Range	16 – 257	142–552	44.428	0.000*
	Mean ± SD	92.93 ± 66.19	310.05 ± 129.77		
Serum AST (U/L)	Range	13–280	110–639	29.231	0.000*
	Mean ± SD	70.15 ± 58.72	238.65 ± 126.41		
Total Serum Bilirubin (mg/dl)	Range	0.50 – 1.58	0.70 – 1.50	0.340	0.563
	Mean ± SD	0.87 ± 0.28	0.92 ± 0.25		
Direct Bilirubin (mg/dl)	Range	0.12 – 0.52	0.14 – 0.60	0.000	1.000
	Mean ± SD	0.31 ± 0.11	0.31 ± 0.14		
Indirect Bilirubin (mg/dl)	Range	0.28 – 1.11	0.48 – 1.00	0.840	0.365
	Mean ± SD	0.56 ± 0.22	0.61 ± 0.17		
Alkaline phosphatase (U/L)	Range	357–607	383–924	1.953	0.017*
	Mean ± SD	456.65 ± 74.08	609.05 ± 105.42		
Albumin (g/dl)	Range	3.3 – 5.2	3.5 – 4.7	1.322	0.257
	Mean ± SD	4.3 ± 0.6	4.11 ± 0.43		
Total protein (g/dl)	Range	6–8.7	5.7 – 8.4	3.725	0.061
	Mean ± SD	7.55 ± 0.78	7.09 ± 0.75		
Albumin/Globulin ratio	Range	1–1.9	1.1 – 1.6	0.471	0.497
	Mean ± SD	1.35 ± 0.26	1.40 ± 0.14		
Prothrombin time (seconds)	Range	12–20.8	18.5 – 21.6	20.923	0.001*
	Mean ± SD	16.75 ± 2.93	19.95 ± 1.10		
Prothrombin activity (%)	Range	63 – 100	60 – 80	5.926	0.020*
	Mean ± SD	77.65 ± 12.83	69.7 ± 6.97		

* Significant P value <0.05.

Liver function is the main value of daily serum levels of different parameters of liver functions determined for seven successive days after giving each methotrexate dose.

**Table 4:** Comparison between studied groups as regard kidney functions before and after chemotherapy.

Before chemotherapy		Group I (No=40)	Group II (No=40)	t. test	P. value
Urea (mg/dl)	Range	13–62	13–69	0.281	0.599
	Mean ± SD	32.75 ±12.52	30.55 ± 13.71		
Creatinine (mg/dl)	Range	0.4 – 1.2	0.5 – 1.1	3.954	0. 54
	Mean ± SD	0.72 ± 0.18	0.68 ± 0.12		
Blood urea nitrogen (mgldl)	Range	9–29	8–27	2.21	0.64
	Mean ± SD	22.11 ± 3.21	21.56 ± 2.77		
Serum Cystatin C (ng/ml)	Range	600–800	612–799	4.61	0.87
	Mean ± SD	671 ± 142	678 ± 139		
Urinary NAG (ng/ml)	Range	6.2–9.1	6.4–9	2.42	0.54
	Mean ± SD	7.12 ± 1.9	7.25 ± 1.7		
	Mean ± SD				
**After chemotherapy**		**Group I (No=40)**	**Group II (No=40)**	**t. test**	**P. value**
Urea (mg/dl)	Range	12–42	15–41	0.683	0.049^*^
	Mean ± SD	21.35 ± 6.92	33.40 ± 8.67		
Creatinine (mg/dl)	Range	0.4 – 1	0.4 – 1.4	0.932	0.034^*^
	Mean ± SD	0.56 ± 0.20	0.85 ± 0.18		
Blood urea nitrogen (mg/dl)	Range	5–19	10–31	0.876	0.022^*^
	Mean ± SD	16.39 ± 3.45	24.98 ± 3.67		
Serum Cystatin C (ng/ml)	Range	900–1600	1300–3900	1.23	0.042^*^
	Mean ± SD	1400±230	2300±640		
Urinary NAG (ng/ml)	Range	8.5–13.4	12 – 24	3.24	0.027^*^
	Mean ± SD	10.3±2.6	18.3±5.4		

NAG = acetyl-beta-D-glucosaminidase.

**Table 5:** Effect of Silymarin use on continuation of chemotherapy.

	Group I (No=40)	Group II (No=40)	X^2^	P. value
Dose continuation	30	20	1.673	0.043*
Dose reduction †	9	14		
Stopping of chemotherapy†	1	6		

* Significant.

† Persistent elevation of liver enzymes more than two folds for one-month warrants a reduction or discontinuation of methotrexate therapy.^13^
